# Regeneration and transformation of *Crambe abyssinica*

**DOI:** 10.1186/s12870-014-0235-1

**Published:** 2014-09-03

**Authors:** Weicong Qi, Iris EM Tinnenbroek-Capel, Jan G Schaart, Bangquan Huang, Jihua Cheng, Richard GF Visser, Eibertus N Van Loo, Frans A Krens

**Affiliations:** Wageningen UR Plant Breeding, Wageningen University and Research Centre, PO Box 386, 6700 AJ Wageningen, the Netherlands; Biotechnology, Jiangsu Academy of Agriculture Science, Zip code 210014 Nanjing City, China; College of Life Science, Hubei University, Zip code 430062 Wuhan City, China; Wuhan Botanical Garden, Chinese Academy of Sciences, Zip code 430074 Moshan, Wuchang Wuhan, China

**Keywords:** Crambe, Oilseed crop, Propagation, Regeneration, Genetic modification, Meristematic tissue, Efficiency

## Abstract

**Background:**

*Crambe abyssinica* (crambe) is a non-food oil seed crop. Its seed oil is widely used in the chemical industry because of the high erucic acid content. Furthermore, it is a potential platform for various feedstock oils for industrial uses based on genetic modification. Here, we describe the development of a series of protocols for all steps required in the process of generating genetically modified crambe.

**Results:**

Different explant types from crambe seedlings were tested for shoot regeneration using different hormone-combinations. Cotyledonary nodes on basic medium with 0.5 μM NAA and 2.2 μM BAP gave the highest regeneration percentages. For propagation by tissue culture, explants of stems, petioles, leaves and axillary buds of *in vitro* plantlets were tested using the optimized medium. Axillary buds showed the highest shoot proliferation efficiency. Cotyledonary nodes were used to test the proper concentration of kanamycin for selection of transformation events, and 10 to 25 mg · L^-1^ were identified as effective. The cotyledonary nodes and cotyledons from 7-day-old seedlings were used in *Agrobacterium*-mediated transformations with two kinds of selection strategies, shifting or consistent. Using the shifting selection method (10 mg · L^-1^ kanamycin, 25 mg · L^-1^, then back to 10 mg · L^-1^) cotyledonary nodes gave 10% transformation frequency, and cotyledons 4%, while with the consistent method (25 mg · L^-1^) lower frequencies were found, 1% for cotyledonary nodes and 0% for cotyledons). Later, *in vitro* plant axillary buds were tried as explants for transformation, however, transformation frequency was low ranging from 0.5 to 2%. Overall, testing six different vectors and two kinds of *Agrobacterium* strains, the average transformation frequency using the shifting method was 4.4%. Determining T-DNA insertion numbers by Southern blotting showed that approximately 50% of the transgenic lines had a single-copy insertion.

**Conclusions:**

Present research revealed the potential of using crambe meristematic tissue for genetic transformation and *in vitro* propagation. The most efficient method of transformation used cotyledonary node explants from 7-days-old seedlings with a shifting kanamycin selection. Meristematic tissues (cotyledonary node or axillary bud) had the highest ability for shoot proliferation. Single-copy T-DNA insert lines could be efficiently and reproducibly generated.

**Electronic supplementary material:**

The online version of this article (doi:10.1186/s12870-014-0235-1) contains supplementary material, which is available to authorized users.

## Background

The industrial oilseed crop *Crambe abyssincia* (crambe) is a non-food oilseed crop from the *Brassicaceae* family, which includes crops such as rapeseed (canola and industrial rapeseed oil) and mustard [1]. Because crambe is a specific non-food crop, its production and processing chain has no overlap with the food production chain. As an oil crop, 33% to 39% of the whole crambe seed including the pod consists of oil of which 55% to 63% is erucic acid (C22:1) [2,3]. Erucic acid and its derivative erucamide [4] are widely used in industry and determine largely the high value of the crambe oil. Its erucic acid content is higher than that in the oil of most other *Brassicaceae* species, including high erucic acid rapeseed (HEAR) [5]. Currently, the main production of erucic acid is still from HEAR, which might present, however, some potential risks. These potential risks reside in intermixing of industrial HEAR with low erucic acid rapeseed (LEAR) which is used for edible oil. Due to their intrinsic different utilities, cross-contamination between them must be strictly avoided, because it will not only decrease both their economical values (qualities), but it might also potentially threaten food security. The amount of erucic acid allowed in food oil has to be lower than 5% (w/w, Council Directive 76/621/EEC). To avoid such problems, crambe has been considered to replace HEAR, because it cannot cross with *Brassica napus* or *B. campestris* (prefertilization barriers) and only very rarely with *B. juncea* (depending on the cultivar, 0 - 2.1% max) [6]. However, the major obstacle for this replacement is that crambe has much lower yields than rapeseed, and hence economically cannot compete with HEAR. Addressing this disadvantage of crambe, the European Union funded the 7^th^ Frame Work Project ‘Industrial Crops producing added value Oil for Novel chemicals’ (ICON) which was planned to overcome the constraints by raising its erucic acid content through genetic modification (GM). Higher erucic acid contents can reduce the total production costs including downstream processing of erucic acid, and accordingly increase profits. Furthermore, another target of ICON was to change not only levels but also the composition of crambe oil by GM [7].

The establishment of *Agrobacterium tumefaciens-*mediated genetic modification (GM) [8] in plants has changed plant science studies remarkably. It brought new strategies and methods to molecular breeding as part of modern crop breeding research. After the first GM crop, the Flavr Savr™ tomato, which carried an artificial antisense gene copy targeted against polygalacturonase in order to prevent premature fruit softening, was put on the market in the USA in 1994 [9], other GM crops have been commercialized around the world in the past 20 years. In 2011 the area of GM crop cultivation was around 160 million hectares [10]. However, in spite of this success, there are still many regions in the world, e.g. the European Union, where the policies against GM food crops are very strict. There has been a concern of GM crops threatening people’s health and the ecosystem, which cannot be ignored. Therefore, worldwide acceptance of GM food is far from being a reality. GM crops for industrial feedstock production, such as crambe, might be more easily accepted by consumers.

To achieve the goals of ICON mentioned above, it is important to have efficient transformation and *in vitro* regeneration/propagation protocols available for crambe. Here, we describe the development of a series of protocols for all steps that are required in the process to obtain GM crambe. It includes *in vitro* tissue regeneration, micro-propagation, selection and *Agrobacterium*-mediated transformation.

## Results

### Shoot regeneration from seedling explants

**Figure 1:**
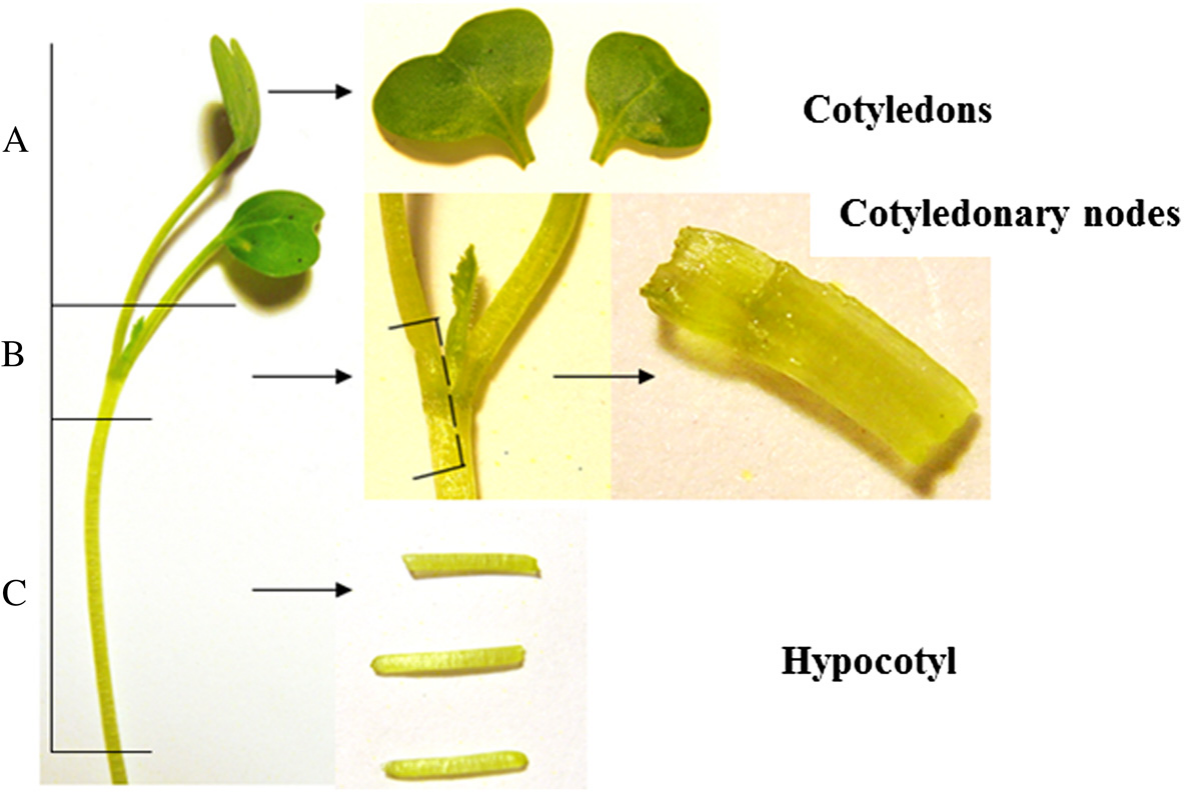
**Cotyledon, cotyledonary node and hypocotyl explants from a 7-day-old crambe seedling.** The parts of the seedling marked **A, B** and **C** are cotyledons, cotyledonary nodes and hypocotyls respectively. When cutting the cotyledonary node explants, extra care is taken to remove the apical bud.

Table 1 shows callus formation and direct shoot regeneration (DSR) and indirect shoot regeneration (ISR) of cotyledon explants (Figure 1) with different hormone combinations and gelling agents after four weeks of culture. Although cotyledons formed callus on all hormone combinations in media solidified with Microagar, no shoot regeneration occurred after seven weeks of culture with one exception, while the explants on Phytoblend-solidified media did show shoot regeneration. The shoot regeneration of cotyledons mostly took place indirectly from the callus. DSR happened only on Phytoblend medium with 0.5 μM NAA and 2.2 μM BAP at a low percentage. The percentage of indirect shoot regeneration from callus generally decreased with increasing BAP concentrations. The results of two-way ANOVA statistical analyses showed that 1) the explants on Phytoblend medium gave significantly (p < 0.01) more ISR than those on Microagar medium; 2) among the media with Phytoblend, the BAP concentration significantly influenced the ISR (p < 0.05), but the NAA did not; 3) neither gelling agent nor hormones had an effect on the DSR.

**Table 1:** **Response of crambe cotyledon explants to different hormone combinations on media with Microagar or Phytoblend**

**Microagar**	**NAA 0**			**NAA 0.5**			**NAA 5**		
	**CI**	**DSR**	**ISR**	**CI**	**DSR**	**ISR**	**CI**	**DSR**	**ISR**
BAP 0	100%	0%	0%						
BAP 0.44				100%	0%	0%	100%	0%	0%
BAP 2.2				100%	0%	0%	95%	0%	0%
BAP 4.4				100%	0%	0%	100%	0%	8%
BAP 22				100%	0%	0%	100%	0%	0%
**Phytoblend**	**NAA 0.5**			**NAA 2.5**			**NAA 5**		
	**CI**	**DSR**	**ISR**	**CI**	**DSR**	**ISR**	**CI**	**DSR**	**ISR**
BAP 2.2	55%	3%	50%	98%	0%	48%	100%	0%	53%
BAP 4.4	95%	0%	35%	100%	0%	45%	100%	0%	58%
BAP 22	30%	0%	35%	80%	0%	13%	48%	0%	20%

Note: hormone concentrations are in μM; CI, callus induction; DSR, direct shoot regeneration i.e. from differentiated tissue; ISR, indirect shoot regeneration. i.e. from callus formed on explant tissue.

In comparison with cotyledons, the cotyledonary node explants (Figure 1; Table 2) gave less callus and ISR but more DSR, varying from 23% to 98% on medium with Microagar. With Phytoblend as gelling agent, the explants gave extremely high levels of DSR among all treatments and the efficiencies ranged from 50% to 100%. The combination of 0.5 μM NAA with Phytoblend showed more ISR than the other combinations. The Phytoblend medium with 0.5 μM NAA and 2.2 μM BAP had explants with 100% DSR and 15% ISR, which was the best. In the Phytoblend media, the frequency of ISR decreased when BAP and NAA concentrations were increased. According to the two-way ANOVA analyses, the conclusions are 1) in the Microagar media, the NAA concentration influenced the DSR and ISR significantly, negative for DSR but positive for ISR; 2) in the Phytoblend medium, neither NAA nor BAP influenced the direct shoot-regeneration; 3) within Phytoblend media, the NAA concentration significantly affected the ISR (p < 0.05).

**Table 2:** **Response of crambe cotyledonary node explants to different hormone combinations on media with Microagar or Phytoblend**

**Microagar**	**NAA 0**			**NAA 0.5**			**NAA 5**		
	**CI**	**DSR**	**ISR**	**CI**	**DSR**	**ISR**	**CI**	**DSR**	**ISR**
BAP 0	0%	0%	0%						
BAP 0.44				25%	90%	0%	78%	53%	10%
BAP 2.2				48%	98%	0%	85%	23%	5%
BAP 4.4				30%	85%	3%	57%	73%	7%
BAP 22				7%	90%	0%	83%	33%	5%
**Phytoblend**	**NAA 0.5**			**NAA 2.5**			**NAA 5**		
	**CI**	**DSR**	**ISR**	**CI**	**DSR**	**ISR**	**CI**	**DSR**	**ISR**
BAP 2.2	35%	100%	15%	50%	100%	3%	45%	100%	3%
BAP 4.4	35%	98%	8%	53%	95%	0%	58%	100%	0%
BAP 22	15%	100%	3%	35%	100%	0%	8%	50%	0%

Note: hormone concentrations are in μM; CI, callus induction; DSR, direct shoot regeneration i.e. from differentiated tissue; ISR, indirect shoot regeneration. i.e. from callus formed on explant tissue.

Hypocotyl explants (Figure 1, Additional file 1) also showed a lot of callus formation on media with Microagar plus 0.5 μM NAA, varying from 93% to 100%; fewer calli were formed on media with 5 μM NAA. DSR and ISR were rarely observed on the media with Microagar, 2% DSR on 0.5 μM NAA + 0.44 μM BAP and 2% ISR on 0.5 μM NAA + 4.4 μM BAP and on 5 μM NAA + 2.2 μM BAP. Using Phytoblend, less callus induction was observed on 0.5 μM NAA than on 2.5 or 5 μM NAA. DSR occurred only on medium with 2.5 μM NAA and 22 μM BAP at 3%. ISR was found at 3% on the combinations 0.5 μM NAA + 4.4 μM BAP, 2.5 μM NAA + 2.2 or 4.4 μM BAP. According to the result of two-way ANOVA analyses, neither gelling agent nor hormone had a significant effect on DSR or ISR.

In summary, according to the results above, the optimal medium for crambe shoot-regeneration contains a hormone-combination of 0.5 μM NAA and 2.2 μM BAP using Phytoblend as gelling agent (at 8 g · L^-1^), using as a standard full MS, with 20 g · L^-1^ sucrose and 2 mg · L^-1^ AgNO_3_. Cotyledons had the highest ISR frequency, and cotyledonary nodes had the highest DSR frequency and moderate ISR, while explants from hypocotyls showed very limited DSR and ISR.

### Shoot forming capacity of explant types from in vitro grown plants

**Figure 2:**
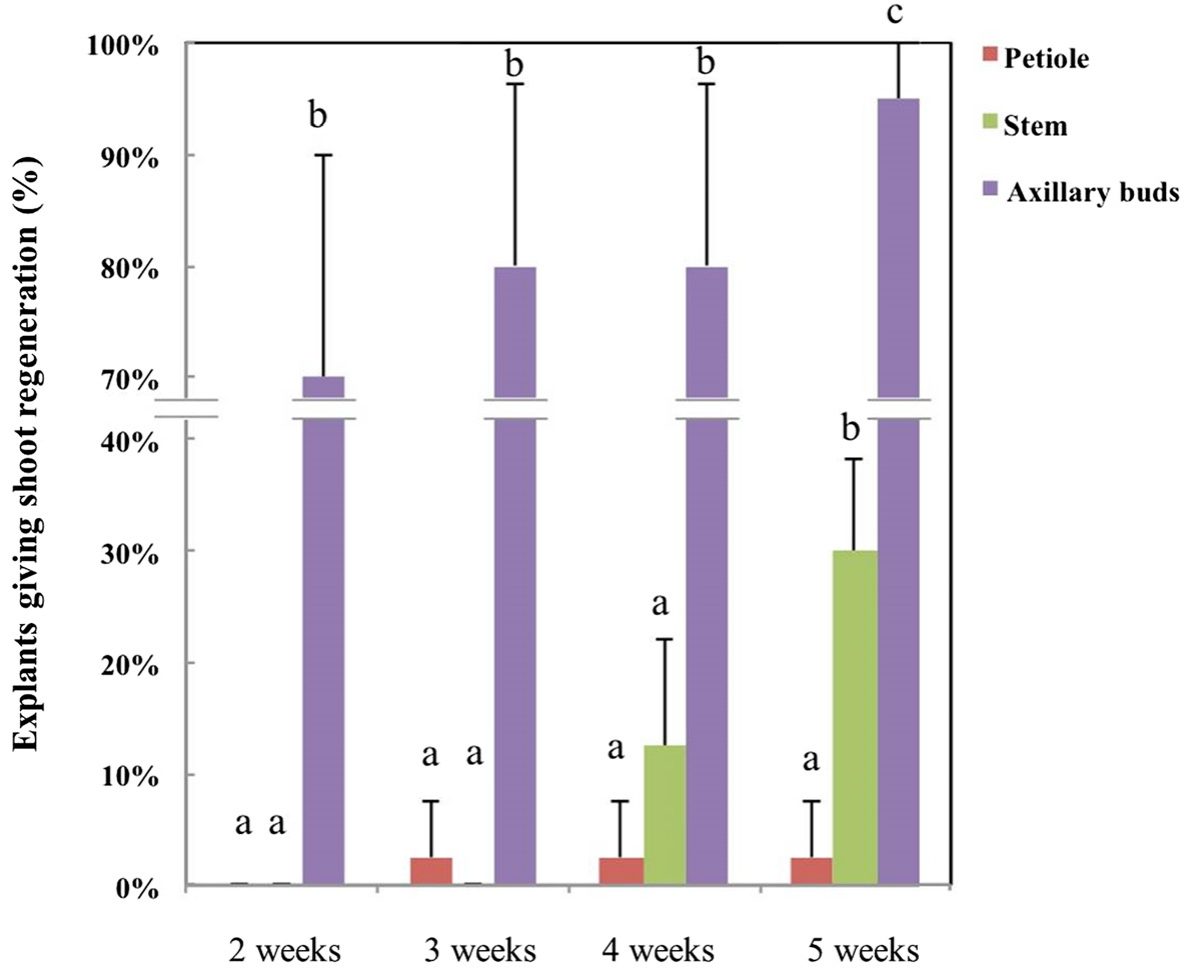
**Shoot regeneration at different time-points of specific explant types from crambe** ***in vitro*** **plants.** Regeneration was scored at different time intervals for four different explant types. The letters above the error bars showed the significant differences analysed by one-way ANOVA. No regeneration was ever observed from leaf explants, so it is not included in the figure.

*In vitro* multiplication of shoots is important for acquiring and maintaining homogeneous starting material for experiments or for clonal propagation of generated GM lines. Here both *de novo* adventitious shoots formation as well as outgrowth of existing meristems were considered. The results of testing the shoot forming capacity of explants derived from established *in vitro* seedlings are shown in Figure 2 in which the direct and indirect shoot regeneration numbers were counted together. *In vitro* leaf parts gave no shoot formation at all on the pre-selected regeneration medium, but only callus. The petioles had a very limited shoot forming capacity of 2.5%. Stem explants showed a moderate frequency of shoot generation at 30%, which was all indirectly from callus and only the explants of axillary buds reached a high level of shoot proliferation. After two weeks, 70% of the cultured axillary bud explants (60 in total) showed shoot formation, which was mostly direct. After five weeks, a shoot formation percentage of 95% was observed; regeneration was both direct (outgrowth of pre-existing meristematic tissue) and indirect (adventitious shoot formation). The amount of regenerating shoots from an individual axillary bud explant varied widely from 0 to more than 10, while on average one explant yielded 6 regenerating shoots. In this whole procedure, axillary buds always gave the highest shoot multiplication rate (significant by one-way ANOVA test; p < 0.01).

### Testing selection for transformants

The results of testing kanamycin treatments on wild-type (WT) crambe seedling explants from cv. ‘Galactica’ are shown in Additional file 2. The controls, growing on medium without kanamycin for four weeks, showed vigorous growth and callus and shoot development for all three explant types tested. On medium with 10 mg · L^-1^ kanamycin, the number of surviving explants (staying green and with green regenerating shoots) already was significantly lower compared to the controls, as well as the callus formation and shoot regeneration, but still some explants with green regenerating shoot clusters were left. Kanamycin at a concentration of 25 and 50 mg · L^-1^, completely inhibited shoot regeneration from all types of explants.

### Transformation

**Figure 3:**
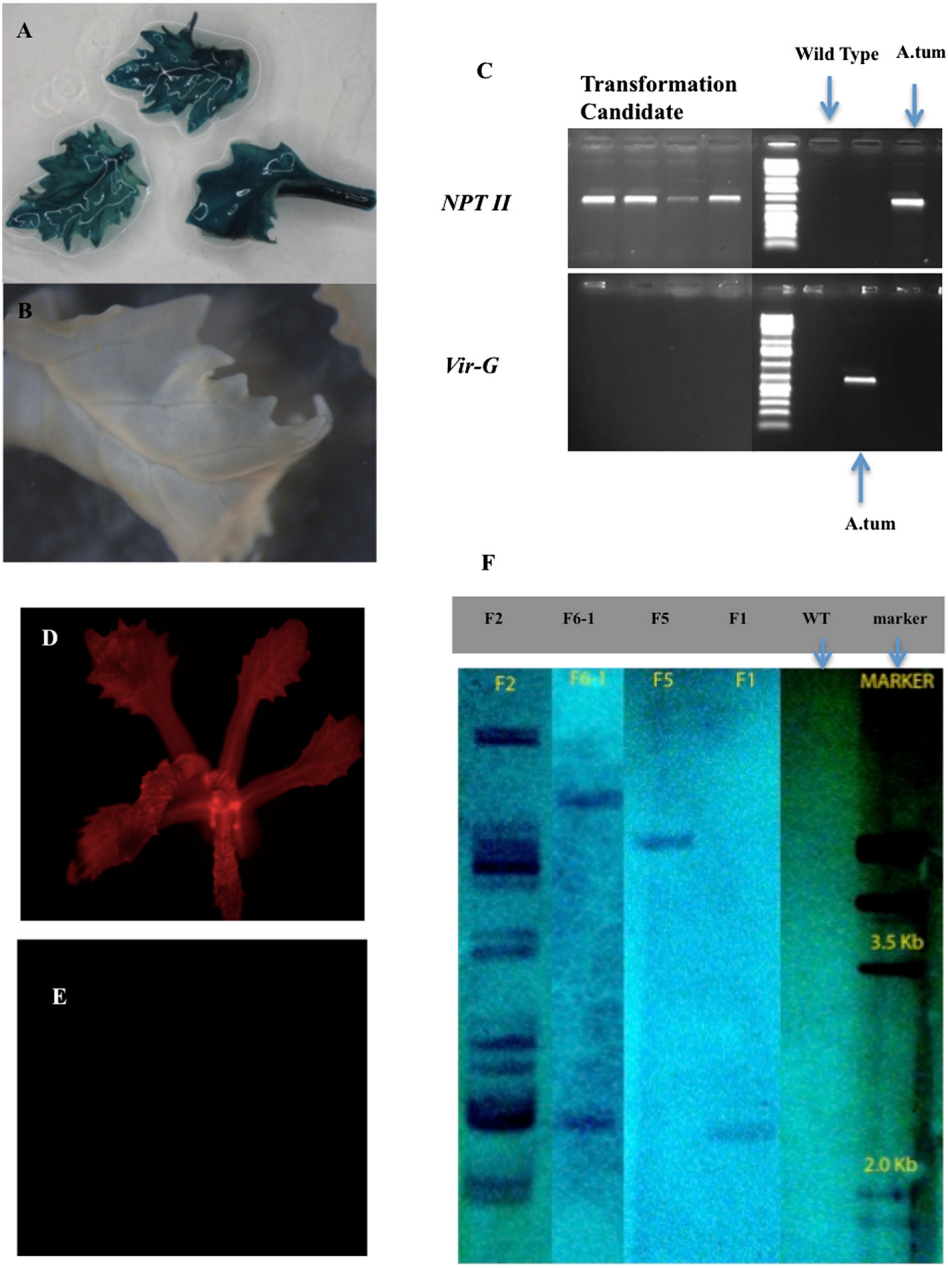
**Histochemical and molecular evidence for the transgenic nature of putative genetically modified crambe shoots. A**, GUS staining of leaf samples from a candidate transformant obtained from cotyledonary node explants inoculated by AGL0 (pJS-M14) with a shifting selection scheme; **B**, GUS staining of a leaf sample from the WT control; **C**, PCR using either primers for *NPTII* (upper panel) or *vir-*G (lower panel). DNA samples from four randomly chosen candidate transformants obtained from cotyledonary node explants inoculated by AGL0 (pJS-M14) with a shifting selection scheme. DNA from wild type (WT) crambe was used as negative control and *Agrobacterium* as positive control. The fluorescent shoot in panel **D** was obtained from the transformation of axillary bud explants with AGL1 carrying pBinGlyRed (AscI), while panel **E** shows the absence of fluorescence in a regenerated shoot of WT. Panel **F** showed the Southern blotting analysis of transformants obtained from cotyledonary node explant based transformation protocol with a shifting selection scheme. DNA samples from four transgenic candidates (coded as F2, F6-1, F5 and F1 from left to right) of pBinGlyRed (AscI) transformation are presented. The restriction enzyme was *Dra*I and the *NPTII* probe was generated using the same primers as used for PCR. Next to the molecular weight marker lane control DNA from WT crambe is loaded. The lower size limit of fragments to be visualized is 2.3 kb.

As displayed in Table 3, cotyledons and cotyledonary node explants, with two methods of selection (shifting: selection on 10 mg · L^-1^ kanamycin for the first two weeks, then followed by four weeks selection on 25 mg · L^-1^ kanamycin, followed by further selection for at least four weeks on 10 mg · L^-1^ kanamycin again; consistent: the kanamycin concentration was continuously high at 25 mg · L^-1^) and two hormone combinations (A: 0.5 uM NAA + 2.2 uM BAP; B: 2.5 uM NAA + 2.2 uM BAP) were used for transformation, and the explants were tested for GUS activity after eight weeks of selection after *Agrobacterium* inoculation. GUS-positive shoots were detected on cotyledonary nodes on both media with shifting selection; only one explant produced GUS + shoots using the consistent selection scheme. The highest numbers of shoots showing GUS staining and being PCR positive (Figure 3A, B and C) came from explants on medium A with shifting selection. For cotyledons, GUS-positive shoots were only obtained from explants on media A, and shifting selection showed a higher frequency than the consistent selection. Summarizing, within these combinations, the highest percentage of GUS positive shoots was 10% for cotyledonary nodes from medium A with shifting selection, more GUS-positive shoots were observed with the shifting selection method compared to consistent selection and explants on medium A gave higher rates of transformation for both cotyledonary nodes and cotyledons. Lastly, cotyledonary nodes gave more positive shoots then cotyledons. It was found that not all plants that survived kanamycin selection for eight weeks proved to be GUS positive.

**Table 3:** **Effect of different explant types and selection methods on transformation frequencies in crambe**

**Explant type**	**Medium**	**Selection**	**No. of explants**	**No. of explants with regenerating shoots**	**No. of explants with GUS + regenerating shoots**	**Transformation frequency**
Cotyledonary node	A	Shifting	111	17	11	10%
		Consistent	116	1	1	1%
	B	Shifting	114	16	4	4%
		Consistent	110	0	0	0%
Cotyledon	A	Shifting	106	4	4	4%
		Consistent	104	1	1	1%
	B	Shifting	110	0	0	0%
		Consistent	98	0	0	0%

Note: transformation frequencies are calculated as the percentage of explants with GUS + shoots per total number of explants. Selection is on kanamycin; shifting: 10 mg.L^-1^ → 25 → 10; consistent: 25 continuously.

### Transformation of axillary bud explants

Axillary buds of *in vitro* grown plants could potentially be used as explants for inoculation. To test their suitability some preliminary transformation experiments were done in which different vectors were used as well as different selection schemes. The numbers of surviving, green shoots were scored after 20 to 30 weeks of selection, which were subsequently screened for GUS activity or DsRed fluorescence. The results are summarized in Table 4. Transformation events were obtained, however, at lower frequencies when compared to cotyledonary node explants. The highest frequency of transformed shoots (2.0%) was obtained when the kanamycin shifting selection method with 10 mg · L^-1^ two weeks, followed by 20 mg · L^-1^ for four weeks, and then back to 10 mg · L^-1^ was used. The experiment with a slightly higher kanamycin concentration in the second period (25 mg · L^-1^) yielded a low transformation rate of 0.5%. Consistent selection with 10 mg · L^-1^ required a longer selection period of 30 weeks. A regenerated transgenic shoot from the transformation with pBinGlyRed (AscI) is shown in Figure 3D (control in Figure 3E). All putative transgenic shoots were confirmed by PCR for the presence of *NPTII* gene.

**Table 4:** **Transformation frequencies using axillary buds from** ***in vitro*** **grown crambe plants**

**Experiment ID**	**Selection**	**Vector**	**Frequency**
Tr11-05	Consistent 10 mg · L^-1^	pBinGlyRed (AscI)	1.6%
Tr12-01	Shifting 10 → 25 → 10 mg · L^-1^	pJS-M14	0.5%
	Shifting 10 → 20 → 10 mg · L^-1^		2.0%

Note: the success of transformation was verified by PCR, GUS staining or fluorescence. Selection is on kanamycin.

### Differences in frequencies of cotyledonary node explant based transformation with different binary vectors

**Figure 4:**
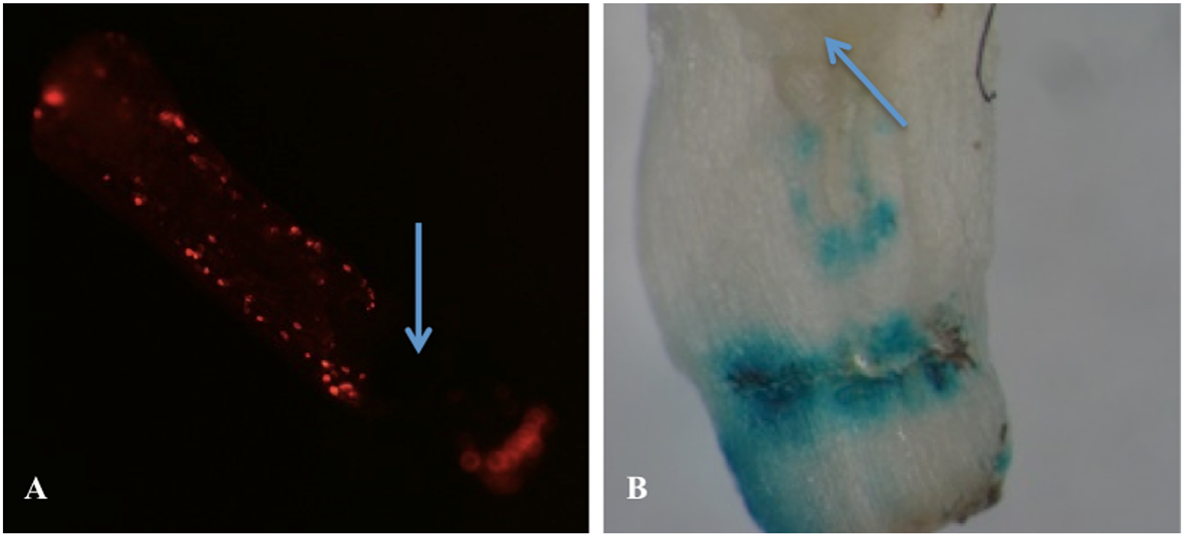
**Location of cells in crambe cotyledonary node explants showing gene uptake two days after co-cultivation. A**, DsRed fluorescence in a cotyledonary node explant two days after co-cultivation with AGL1 (*pBinGlyRed* (AscI)); **B**, GUS staining of a cotyledonary node explant two days after co-cultivation with AGL0 (pJS-M14); Arrows show the meristematic zones in the explants. Note the absence of gene transfer in these zones as judged by the absence of colour.

Finally, the method using cotyledonary node explants from 7-days-old seedling and the shifting selection (10 → 25 → 10 mg · L^-1^ kanamycin) was used as the standard protocol for crambe transformation. In total, 12 different transformation experiments, performed at different dates or using different vector/strain combinations, using this protocol are listed in Table 5. The binary vectors used all contained the *NPTII* gene as selectable marker but differed in backbone (origin) and reporter genes or genes-of-interest involved in the regulation of seed oil composition. The vectors were present in two comparable *Agrobacterium* strains, either AGL0 or AGL1. Supervirulent AGL0 is an EHA101 derivative (C58 pToBo542) with the T-region deleted; AGL1, is derived from AGL0 and Rec^-^ [11]. All of the transformation events were confirmed by PCR, and representative samples were proven transgenic by Southern blotting (Figure 3F). The average frequency was 4.4%, after an average selection period of 20 weeks. The pHan2 in AGL0, and pBinGlyRed (KpnI) in AGL1 showed relatively low frequencies, i.e. lower than 2%. The variation from one experiment to the other is also evident for pJS-M14 and pBinGlyRed (AscI). These two vectors were also used to check where gene transfer was accomplished by *Agrobacterium* on cotyledonary node explants monitoring (transient) gene expression of GUS and DsRed, two days after cocultivation. It was found that the meristematic areas of the explants were almost completely devoid of gene transfer events (Figure 4). The T-DNA insertion number of the T0 GM crambe lines confirmed by Southern blotting was shown to range from single insert (49%), low insert number (2 to 3, 35%) to multiple inserts (>3, 16%) and was independent of the strain or binary vector that was used.

**Table 5:** **Transformation frequencies using different vectors in two** ***Agrobacterium*** **strains and crambe cotyledonary node explants with the optimized protocol**

**Experiment code**	**Vector**	**Vector size/bp**	***A.tum***	**Period/week**	**Percentage of transformation**
Tr08-01	pJS-M14	21,071	AGL0	20	9.9%
Tr08-02	pJS-M14	21,071	AGL0	19	4.4%
Tr12-01	pJS-M14	21,071	AGL0	20	4.0%
T11-03	pJS-M14	21,071	AGL0	19	7.5%
T10-01	pHan2	16,783	AGL0	24	1.5%
T09-10	pBinGlyRed (Asc I)	14,937	AGL1	19	4.0%
T10-02	pBinGlyRed (Asc I)	14,937	AGL1	16	3.8%
T10-03	pBinGlyRed (Asc I)	14,937	AGL1	18	5.8%
T09-10	pBinGlyRed (Kpn I)	17,662	AGL1	19	1.0%
T10-04	pBinGlyRed (Kpn I)	17,662	AGL1	18	2.0%
T12-02	pHellsgate LPAT2-RNAi	15,392	AGL1	20	2.5%
Tr12-05	pWatergate-3G	23,746	AGL1	20	2.3%

Note: pJS-M14 carries two reporter genes, i.e. *gus* and *gfp*; pBinGlyRed (AscI) carries DsRed as reporter gene. In total 2400 explants were inoculated with *Agrobacterium*.

## Discussion

In the present research, a series of protocols for crambe *in vitro* regeneration, selection and transformation are described. The research presented here showed that the most efficient regeneration was obtained from cotyledonary nodes isolated from seedlings and axillary buds taken from *in vitro* grown plantlets as explant types. A combination of the plant growth regulators NAA (0.5 uM) and BAP (2.2 uM) was shown to be optimal in standard MS20 (Full MS, 20 g · L^-1^ sucrose and 2 mg · L^-1^ AgNO_3_, pH 5.8). Furthermore, the gelling agent used was found to play an important role in determining regeneration efficiency, with Phytoblend performing better than Microagar. Comparing the regeneration efficiencies from hypocotyls to the ones obtained in this study from axillary buds and cotyledonary nodes, the latter were higher, however, the mode of regeneration might be different. Cotyledonary nodes and axillary buds contain meristematic tissue. This might explain why they showed more direct shoot regeneration than other explant types (leaf, petiole, cotyledon, stem and hypocotyl). Meristem outgrowth, especially regeneration from axillary buds is a useful tool for *in vitro* plant propagation [12]. *In vitro* propagation can be extremely useful in multiplying any putative transgenic crambe events, generating sufficient clonal material for either DNA isolation or rooting and transfer of T0 lines into the greenhouse. Cloned individuals can be subjected to further antibiotic selection, thereby decreasing the chance of getting escapes or chimeras. The latter is important because explant types containing meristematic tissue are considered prone to producing chimeric plants. In our experiments, the clonally propagated putative transgenic lines were subjected to renewed selection on 10 mg · L^-1^ kanamycin for approximately three weeks, and when the entire clone stayed green (entire regenerating cluster), the candidate was considered transgenic. If some parts bleached, it meant that the original shoot was either an escape or a chimera, for which longer selection was needed. However, prolonging the selection period does not guarantee elimination of all chimeric tissue. To exclude totally any chance of working with chimeric plants, going through a seed phase is considered to be essential in order to get stable, homogeneous transgenic crambe plant lines. Two protocols of crambe transformation have been reported earlier, one by [13] based on hygromycin selection (20 mg · L^-1^), and another by [14] based on kanamycin selection (25 mg · L^-1^). Both used hypocotyls as explants, which in our hands yielded very low transformation efficiencies. T-DNA insert copy numbers seemed to be higher using hypocotyls. For efficient plant transformation protocols, the choice of the proper selection agent and the optimal concentration to use is crucial. We tested killing curves of kanamycin for cotyledonary node explants and determined the concentration at which regeneration would be totally abolished, preferably without complete killing of the original explant. We also showed the effect of different exposure schemes, varying from a specific concentration continuously throughout the entire selection to shifting from one concentration to another higher one later and back to the original concentration again. The results indicated that the shifting treatments gave better results for transformation efficiency than a consistent one in case of kanamycin selection. The shift in concentrations originated from the earlier observation, that in wild type material 10 mg · L^-1^ kanamycin still allowed escapes up to a level of 17% after four weeks (Additional file 2), while the consistently high selective pressure of 25 mg · L^-1^ kanamycin proved too harsh as only a very limited number of transformants could be obtained in this way. Dying untransformed cells might negatively influence the viability of transformed cells hampering regeneration. On the other hand at lower concentrations, untransformed cells might benefit from the detoxifying capabilities of transformed cells and survive selection giving rise to escapes or perhaps chimeras. Our research showed that the frequency of escapes at a concentration of 10 mg · L^-1^ was much higher for hypocotyl explants (37%) than for the other explant types tested (19% for cotyledons and 17% for cotyledonary nodes). This could suggest that the tolerance level of hypocotyls to kanamycin was higher than that of other explants. Li et al. [14] developed a protocol for crambe transformation based on hypocotyl explants, using 25 mg · L^-1^ kanamycin selection and obtained a transformation frequency between 1% to 3%. Hence, consistent selection at 25 mg · L^-1^ could be suited for selection in hypocotyls, but not for selection in cotyledons and cotyledonary nodes.

In our transformation experiments, using kanamycin selection and cotyledonary node explants, the transformation frequencies varied from 1 to 10% with an average of 4.4% with respect to the efficiency (Table 4.). Previously, similar cotyledonary node-based protocols have also been used in transformation of sugar beet [15] and caraway [16]. In caraway, the cotyledonary-node transformation method yielded confirmed transformants through a seemingly direct shoot regeneration process. On the other hand for sugar beet, no transformed shoots were obtained from the first series of direct regenerants, but only from later series with or without visible signs of dedifferentiation. In this research, similar to sugar beet, we never found any GUS positive transformants among the directly regenerating shoots from cotyledonary nodes. All the GUS positive and DsRed fluorescing shoots were obtained later after the first, directly regenerating shoots had been removed. During the process of cotyledonary node transformation and regeneration, we observed that all the callus and indirect regenerating shoots were formed in the region around the original meristematic zone, while directly regenerating shoots emerged from this zone itself. This might suggest that the actively growing meristem cells can influence surrounding cells inducing them to differentiate into developing shoots. GUS staining and DsRed fluorescence shortly after co-cultivation demonstrated that some areas on the cotyledonary node explants showed evidence of gene transfer (Figure 4) but not the meristematic zones. Therefore, we believe that the transformed shoots were mostly derived through indirect regeneration, thereby reducing the chance of finding chimeras. Crucial in this phenomenon is whether the regeneration comes form a single cell or from a cluster of multiple cells. The latter cannot be excluded. Although cotyledons gave more indirect shoot regeneration from primary formed callus, there were just too few transgenic events leading to the generation of transgenic shoots. Accordingly, the cotyledonary-node method was chosen as the main protocol for the transformation of crambe. Meristematic tissues have also been used for sunflower [17], cotton [18] and soybean [19] transformation.

The new protocol developed here was applied for transformation with several different vectors. No correlation could be found between vector traits such as size or origin and the transformation frequency, nor was any difference observed between the two *Agrobacterium* strains used (AGL0 and AGL1). Finally, with all vectors used we found T-DNA insert copy numbers ranging from one single copy to more than six (multiple) copies. The chance of acquiring multiple T-DNA insertion lines (51%) was similar to getting single copy-insertion lines (49%).

Although transformation of axillary buds from *in vitro* grown plants showed low efficiencies compared to the cotyledonary nodes, this method might serve as a more convenient alternative method for retransformation using different selection agents or retransformation after generating marker-free plants when seeds for preparing cotyledonary nodes are not yet available. However, further improvements are still needed to make it into a stable and reliable method.

## Conclusions

Present research revealed the possibility of using crambe meristem tissue from seedling or *in vitro* plant, for genetic transformation and *in vitro* propagation. The most efficient method of transformation was based on cotyledonary node explants from 7-days-old seedlings with a shifting kanamycin selection (from 10 mg · L^-1^ to 25 mg · L^-1^, and then back to 10 mg · L^-1^), and it indicated the frequency varied from 1% to 10%. Moreover, in comparison with other kinds of tissue, the meristem tissue (cotyledonary node or axillary bud) had the highest ability for shoot proliferation. According to the experience from performing the transformation protocol with six different vectors and two kinds of Agrobacterium strains, the average frequency was 4.4% with a term as long as 20 weeks. The result of T0 plant T-DNA insertion number determination by Southern blotting showed that, there was a 50/50% chance of having single -or multi-insertion lines.

## Methods

### Plant materials, seed sterilization & germination

Seeds of crambe cv. ‘Galactica’ harvested July 2007 from a seed production field in Wageningen, The Netherlands were used. For sterilization, a gram of hulled crambe seeds were put into a 50 ml centrifuge tube with cap, firstly with 30 ml 70% (v/v) ethanol and shaken for 30 seconds for surface sterilisation. Subsequently, the ethanol was removed, and 30 ml 3% (w/v) NaClO with 300 μl Tween20 was added to the tube. With the cap closed the tube with seeds was put into a water bath at 42.5°C for 20 minutes. After that, the seeds were rinsed with sterile demineralized water four times. Finally, the sterilized seeds were placed onto germination medium consisting of MS salts and vitamins [20], 2% (w/v) sucrose and 0.8% (w/v) Phytoblend at pH 5.8 in a plastic high container (50 seeds/container). The seeds were incubated at 7°C overnight in the dark, followed by placing in the dark at 24°C for three days, and further culture at 24°C in 16 h light (with a light intensity of 33 μmol · m^2^ · s^-1^) and 8 h dark period (16hL/8hD) for another three days (in total, seven days of germination after seed sterilization).

### Optimization of crambe regeneration medium

Three different explant types, cotyledons, cotyledonary nodes and hypocotyls (Figure 1) were isolated from 7-days-old seedlings of wild type (WT) crambe, and inoculated into two series (with gelling agents Microagar or Phytoblend at 8 g · L^-1^) regeneration media with varying combinations of auxin NAA (0, 0.5, 2.5 and 5 μM) and cytokinin BAP (0, 0.44, 2.2, 4.4 and 22 μM) (Table 1 and 2). The basic medium composition was full MS, 2 mg · L^-1^ AgNO_3_ and 20 sucrose, pH 5.8. The explants were transferred to fresh media after four weeks and final regeneration was scored after seven weeks of culture at 24°C with 16hL/8hD. Each combination was tested with at least 40 to 60 explants (20 explants/Petri dish) of each type. The effect of gelling agents and hormone concentrations on callus formation, direct shoot regeneration and indirect shoot regeneration from the explants were evaluated by two-way ANOVA.

### Shoot formation capacity of explant types from in vitro plants

Rooted *in vitro* seedlings of eight-weeks-old were taken for testing the shoot forming potential of explant types, such as leaves, petioles, stems and axillary buds. Here, only one medium was used, i.e. the optimal regeneration medium as determined in the previous section, which was basic medium with 0.5 μM NAA and 2.2 μM BAP. The leaves were cut into square pieces of 1 cm^2^ and both petioles and stems were cut into small pieces with a length of 2 mm. Axillary buds were isolated by cutting stem parts carrying a petiole and leaf, removing the leaf and cutting the petiole at the part where it is connected to the stem; in this way, the axillary bud is at the end of the cut petiole. This part was used as explant and put on the regeneration medium. For each explant type 60 explants (20 explants/Petri dish) were taken and scored.

### Testing selectable agents

As a standard medium, full MS (salts and vitamins) with 2 mg · L^-1^ AgNO_3_, 0.5 μM NAA, 2.2 μM BAP, 20 g · L^-1^ sucrose, and 8 g · L^-1^ Phytoblend at pH 5.8 was used. For testing kanamycin [21], hypocotyls, cotyledons and cotyledonary nodes of 7-day-old WT seedlings were used as explants. All explants were cultured with different kanamycin concentrations (0, 10, 25, 50 mg · L^-1^).

### *Agrobacterium*-mediated transformation via seedling parts based on kanamycin selection

#### Vector and *Agrobacterium* strain

Two vectors were used for testing crambe transformation. One is pJS-M14 based on the pMF1 vector system for generating marker-free transgenic plants [22], in which expression of the reporter genes *GFP* and *GUS* was driven by the apple Rubisco-promotor [23]; resistance to the selectable agent kanamycin is provided by a gene fusion between *NPTII* [24] and *CodA* driven by the 35S CaMV promoter. The vector was transformed into *Agrobacterium* strain AGL0 [11]. The other binary vector is pBinGlyRed (AscI) with *NPTII* and *DsRed* [25] as markers, which were driven by the 35S CaMV promoter and the Cassava Vein Mosaic Virus (CsVMV) promoter [26] respectively. This second plasmid was in *Agrobacterium* strain AGL1 [11].

#### Inoculation with *Agrobacterium* and co-cultivation

From a -80°C *Agrobacterium* stock an aliquot was taken and cultured overnight in 10 ml liquid culture medium (Luria-Bertani < LB > medium + 50 mg · L^-1^ kanamycin + 50 mg · L^-1^ rifampicin) at 28°C with constant shaking. The agrobacteria were collected by centrifugation at 3500-4000 g for 10 min, subsequently the pellet was re-suspended in liquid MS medium with 2% sucrose and 100 μM acetosyringone (AS) till an optical density (600 nm) of 0.4. The cotelydonary node explants (Figure 1) were cut from 7-day-old seedlings in liquid MS20 plus 100 μM AS. After cutting, the cotyledonary node explants were placed in a tea strainer, which was placed in the *Agrobacterium* suspension. After inoculation for 30 minutes, the cotyledonary nodes explants are blotted dry and placed with the curved side (outer side) on co-cultivation medium, MS20 + 100 μM AS + 0.5 μM NAA and 2.2 μM BAP, 2 mg · L^-1^ AgNO_3_ and 8 g · L^-1^ Phytoblend, pH 5.8. The cotyledon explants (Figure 1) were cut in a Petri dish with liquid MS20 containing 100 μM AS. After cutting, the cotyledon explants were also placed in a tea strainer in the *Agrobacterium* suspension. After inoculation the explants were blotted dry and placed with the cut side on co-cultivation medium. The Petri dishes were placed for 2 days at 24°C and 16hL/8hD under dimmed light (a cheese cotton cloth on top of the Petri dishes) before putting them under full light conditions.

#### Selection

After co-cultivation the explants were transferred to selection medium containing cefotaxim (200 mg · L^-1^) and timentin (150 mg · L^-1^) to kill the agrobacteria and kanamycin for the selection of the transgenic cells/shoots (20 explants/dish). Petri dishes were closed with leucopor tape and placed at 24°C and 16hL/8hD. The explants were transferred every 2 weeks to fresh selection medium. There were 2 kinds of hormone combinations used in the selection media: Formula A was 0.5 μM NAA + 2.2 μM BAP; formula B was 2.5 μM NAA + 22 μM BAP. Simultaneously, 2 selection regimes were used. Method 1 (shifting) was: selection on 10 mg · L^-1^ kanamycin for the first two weeks, then followed by four weeks selection on 25 mg · L^-1^ kanamycin, followed by further selection for at least four weeks on 10 mg · L^-1^ kanamycin again. In method 2 (consistent) the antibiotic concentration was high at 25 mg · L^-1^ continuously, and the selection period was minimally ten weeks.

#### Rooting and transfer to soil

After selection, green, regenerated shoots were isolated and put onto rooting medium. The rooting medium consisted of MS20 with 0.5 μM NAA and 8 g · L^-1^ Phytoblend. It contained cefotaxim and timentin but no kanamycin. After the *in vitro* shoots had developed adventitious roots, they were moved to the soil for further development. For this, the plants were taken carefully from the tissue culture containers and freed from medium without damaging the roots. The leaves at the bottom and middle part of the plant were removed, leaving the top three or four leaves only. Transferred shoots were covered with a beaker at first to maintain high humidity. The beaker was gradually lifted to allow acclimatization for two to three days and then replaced with a transparent and permeable cover for protecting the plant from insects till they had grown 10 cm high.

#### *Agrobacterium*-mediated transformation via axillary buds

First, *in vitro* plants were prepared by transferring regenerated shoots to rooting medium. After they grew bigger (i.e. longer than 1 cm) with well-developed roots and more than two visible axillary buds, they were considered ready for preparing axillary bud explants. The stem parts with an axillary bud were cut and separated. It was further divided by transversely cutting along the petiole. The petiole side is the axillary bud explant needed for inoculation. These explants were collected and put in a tea strainer, which was submerged in the *Agrobacterium* suspension for a maximum of 30 minutes. After inoculation, the explants were blotted dry before placing them with the cut side on co-cultivation medium (20 explants per dish). The Petri dish was sealed with leucopor. Three selection schemes were used for axillary bud transformation: 1) 10 mg · L^-1^ consistent; 2) shifting I, i.e. 10 mg · L^-1^ for two weeks, 25 mg · L^-1^ for four weeks, and then back to 10 mg · L^-1^; 3) shifting II, i.e. 10 mg · L^-1^ for two weeks, 20 mg · L^-1^ for four weeks, and then back to 10 g · L^-1^. The vectors pBinGlyRed (AscI) in *Agrobacterium* strain AGL1, and pJS-M14 in AGL0 were used to test for transformation.

#### Transformation frequency using different vectors

In further experiments to transform functional, economically important genes into crambe, six different vectors (Table 5) were used. These vectors were present in either *Agrobacterium* strain AGL1 or AGL0. The standard transformation protocol as described earlier was used for these vectors. The selection scheme was the one with a shift in kanamycin concentration, i.e. first two weeks on 10 mg · L^-1^ kanamycin followed by four weeks on 25 mg · L^-1^ cultivation; for the rest of the selection period, the kanamycin selection pressure was kept at 10 mg · L^-1^. Green shoots from regeneration clusters on the explants were cut off after four weeks on selection and placed into regeneration medium including the selective agent directly. These shoots were kept on selection medium at least ten weeks. If at that time they were still not bleached, they were transferred into rooting medium for rooting. After establishment of a rooting system, they were prepared for transfer into soil and to a greenhouse. At this stage material was harvested for DNA isolation for PCR and Southern blotting. Plants in the greenhouse (T0’s) were allowed to self and set seed (T1 seed). All these vectors contained the *NPTII* gene as selectable marker, and in the vector pBinGlyRed (AscI) there is an active non-lethal reporter integrated, the fluorescent protein gene DsRed driven by cassava vein mosaic virus (CsVMV) promoter; in pJS-M14, there are a *GUS* gene and a *GFP* gene present driven by the apple Rubisco promoter.

### Verification of transgenic nature of plants

#### GUS-staining

Histochemical GUS staining of leaves was carried out as described by [27] using a modified staining solution containing 1 mM 5-bromo-4-chloro-3-indolyl β-D- glucuronide (X-gluc) in 50 mM sodium phosphate buffer, pH 7.5, 10 mM ethylenediaminetetraacetic acid (EDTA), 0.5 mM potassium ferricyanide, 5% (w/v) polyvinylpyrrolidone-40 and 0.1% (v/v) Triton X-100. Chlorophyll was removed by washing with 70% (v/v) ethanol.

#### PCR

Genomic DNA was isolated from young leaves of candidate T0 plants with the method described by Aldrich and Cullis [28] but with 1% (w/v) polyvinylpyrrolidone-10 in the DNA extraction buffer. *NPTII* primers were used to demonstrate the presence of the selectable marker gene. *VirG* primers were used for amplifying the *virG* gene [29] from the virulence plasmid to test for the residual contamination by agrobacteria in the plant material. If the result of *virG* was positive, the material cannot be moved to the greenhouse into soil for seed ripening. The primer sequences were: for *NPTII*, forward 5′- TGGGCACAACAGACAATCGGCTGC-3′ and reverse 5′-TGCGAATCGGGAGCGGCGATACCG-3′, while for *virG,* forward 5′-GCCGGGGCGAGACCATAGG-3′ and reverse 5′-CGCACGCGCAAGGCAACC-3′. The expected fragment sizes after amplification are around 686 bp bp for *NPTII* and 606 bp for *virG.* Cycling conditions were 94°C for 1 min, followed by 35 cycles of 94°C (30 sec), 59°C for *nptII* and *virG* (20 sec), and 72°C (30 sec) with a final extension at 72°C for 5 min. for both genes.

#### Southern blotting

Genomic DNA was isolated as described above. The design of the probe and the choice of the appropriate restriction enzyme were based on the sequence of the specific vectors. The labelling system was the DIG-High Prime DNA Labelling and Detection Starter Kit I, Roche. For copy number determination [30], a total of 40 μg of DNA was digested overnight with a restriction enzyme (*Dra*I, *Eco*RI or *Hind*III) and fractionated on a 0.8% (w/v) agarose gel and transferred to Hybond N + membrane (Amersham Biosciences, UK) according to the manufacturer’s recommendations. The membrane was hybridized at 65°C overnight with 20 ng of the labelled probe, targeting *NPTII* or CaMV 35 S promoter using the same primers as for PCR, and washed for 2 times 30 minutes with 0.1 × saline-sodium citrate (SSC) buffer, 0.1% (w/v) SDS at 65°C.

#### Fluorescence microscopy

DsRed fluorescence from the plant material transformed with the construct pBinGlyRed (AscI) was checked using UV fluorescence microscopy (Zeiss, SteREO Discovery.V8 equipped with PentaFluar S fluorescence equipment and PROIR, Lumen 200 illumination system).

## Additional files

Additional file 1:
**Response of hypocotyl explants to different hormone combinations on media with Microagar or Phytoblend.**
Additional file 2:
**Effect of different kanamycin concentrations on regeneration from hypocotyl, cotyledonary node and cotyledon explants.**

## References

[1] White GA, Higgins JJ (1966). Culture of Crambe: A new Industrial Oilseed Crop.

[2] White GA (1975). Distinguishing characteristics of *Crambe abyssinica* and *C hispanica*. Crop Sci.

[3] Rudloff E, Wang Y, Kole C (2011). Crambe. Wild Crop Relatives: Genomic and Breeding Resources.

[4] Ramírez MX, Hirt DE, Wright LL (2001). AFM characterization of surface segregated erucamide and behenamide in linear low density polyethylene film. Nano Lett.

[5] Sahasrabudhe MR (1977). Crismer values and erucic acid contents of rapeseed oils. J Am Oil Chem Soc.

[6] Youping W, Peng L (1998). Intergeneric hybridization between *Brassica* species and *Crambe abyssinica*. Euphytica.

[7] Stymne S, Dyer J (2007). Oil Crop Platforms for Industrial Uses.

[8] Fraley RT, Rogers SG, Horsch RB, Sanders PR, Flick JS, Adams SP, Bittner ML, Brand LA, Fink CL, Fry JS, Galluppi GR, Goldberg SB, Hoffmann NL, Woo SC (1983). Expression of bacterial genes in plant cells. Proc Natl Acad Sci.

[9] Kramer M, Redenbaugh K (1994). Commercialization of a tomato with an antisense polygalacturonase gene: The FLAVR SAVR™ tomato story. Euphytica.

[10] Nábrádi A, Popp J: **Economics of GM crop cultivation.** *Crop Biotech Update* 2011, (June 3 issue):7–19.

[11] Lazo GR, Stein PA, Ludwig RA (1991). A DNA transformation-competent *Arabidopsis* genomic library in *Agrobacterium*. Nat Biotechnol.

[12] Walkey D (1972). Production of apple plantlets from axillary-bud meristems. Can J Plant Sci.

[13] Chhikara S, Dutta I, Paulose B, Jaiwal PK, Dhankher OP (2012). Development of an *Agrobacterium*-mediated stable transformation method for industrial oilseed crop *Crambe abyssinica* ‘BelAnn’. Ind Crop Prod.

[14] Li X, Ahlman A, Yan X, Lindgren H, Zhu L-H (2010). Genetic transformation of the oilseed crop *Crambe abyssinica*. Plant Cell Tiss Org.

[15] Krens FA, Trifonova A, Paul Keizer LC, Hall RD (1996). The effect of exogenously-applied phytohormones on gene transfer efficiency in sugarbeet (*Beta vulgaris* L.). Plant Sci.

[16] Krens FA, Keizer LCP, Capel IEM (1997). Transgenic caraway, *Carum carvi* L.: a model species for metabolic engineering. Plant Cell Rep.

[17] Schrammeijer B, Sijmons P, Elzen PM, Hoekema A (1990). Meristem transformation of sunflower via Agrobacterium. Plant Cell Rep.

[18] Chlan C, Lin J, Cary J, Cleveland T (1995). A procedure for biolistic transformation and regeneration of transgenic cotton from meristematic tissue. Plant Mol Biol Rep.

[19] Christou P, McCabe DE, Martinell BJ, Swain WF (1990). Soybean genetic engineering - commercial production of transgenic plants. Trends Biotechnol.

[20] Murashige T, Skoog F (1962). A Revised medium for rapid growth and bio assays with tobacco tissue cultures. Physiol Plant.

[21] Pestka S, Moldave K, Grossman L (1974). The use of inhibitors in studies of protein synthesis. Methods in Enzymology.

[22] Schaart JG, Krens FA, Pelgrom KTB, Mendes O, Rouwendal GJA (2004). Effective production of marker-free transgenic strawberry plants using inducible site-specific recombination and a bifunctional selectable marker gene. Plant Biotechnol J.

[23] Schaart J, Tinnenbroek-Capel IM, Krens F (2011). Isolation and characterization of strong gene regulatory sequences from apple, *Malus × domestica*. Tree Genet Genomes.

[24] Beck E, Ludwig G, Auerswald EA, Reiss B, Schaller H (1982). Nucleotide sequence and exact localization of the neomycin phosphotransferase gene from transposon Tn5. Gene.

[25] Baird GS, Zacharias DA, Tsien RY (2000). Biochemistry, mutagenesis, and oligomerization of DsRed, a red fluorescent protein from coral. Proc Natl Acad Sci.

[26] Verdaguer B, de Kochko A, Fux C, Beachy R, Fauquet C (1998). Functional organization of the cassava vein mosaic virus (CsVMV) promoter. Plant Mol Biol.

[27] Jefferson R (1987). Assaying chimeric genes in plants: The GUS gene fusion system. Plant Mol Biol Rep.

[28] Aldrich J, Cullis C (1993). RAPD analysis in flax: Optimization of yield and reproducibility using klenTaq 1 DNA polymerase, chelex 100, and gel purification of genomic DNA. Plant Mol Biol Rep.

[29] Stachel SE, Zambryski PC (1986). virA and virG control the plant-induced activation of the T-DNA transfer process of A. tumefaciens. Cell.

[30] Höltke HJ, Ankenbauer W, Mühlegger K, Rein R, Sagner G, Seibl R, Walter T (1995). The Digoxigenin (DIG) system for non-radioactive labeling and detection of nucleic acids-an overview. Cell Mol Biol.

