# Supplementary material for: Regeneration and transformation of Crambe abyssinica
Source: BMC Plant Biol. 2014 Sep 3;14:235. doi: 10.1186/s12870-014-0235-1 (PMC4156612; doi:10.1186/s12870-014-0235-1)
Supplement: Additional file 1: — Response of hypocotyl explants to different hormone combinations on media with Microagar or Phytoblend. [file 12870_2014_235_MOESM1_ESM.docx]

**Additional file 1 Response of hypocotyl explants to different hormone combinations on media with Microagar or Phytoblend**

| Microagar | NAA 0 | | | NAA 0.5 | | | NAA 5 | | |
| --- | --- | --- | --- | --- | --- | --- | --- | --- | --- |
|  | CI | DSR | ISR | CI | DSR | ISR | CI | DSR | ISR |
| BAP 0 | 0% | 0% | 0% |  |  |  |  |  |  |
| BAP 0.44 |  |  |  | 100% | 2% | 0% | 58% | 0% | 0% |
| BAP 2.2 |  |  |  | 100% | 0% | 0% | 87% | 0% | 2% |
| BAP 4.4 |  |  |  | 93% | 0% | 2% | 72% | 0% | 0% |
| BAP 22 |  |  |  | 100% | 0% | 0% | 28% | 0% | 0% |
| Phytoblend | NAA 0.5 | | | NAA 2.5 | | | NAA 5 | | |
|  | CI | DSR | ISR | CI | DSR | ISR | CI | DSR | ISR |
| BAP 2.2 | 88% | 0% | 0% | 100% | 0% | 3% | 100% | 0% | 0% |
| BAP 4.4 | 95% | 0% | 3% | 100% | 0% | 3% | 100% | 0% | 0% |
| BAP 22 | 88% | 0% | 0% | 100% | 3% | 0% | 100% | 0% | 0% |

Note: hormone concentrations are in μM; CI, callus induction; DSR, direct shoot regeneration i.e. from differentiated tissue; ISR, indirect shoot regeneration. i.e. from callus formed on explant tissue.
