# Supplementary material for: Regeneration and transformation of Crambe abyssinica
Source: BMC Plant Biol. 2014 Sep 3;14:235. doi: 10.1186/s12870-014-0235-1 (PMC4156612; doi:10.1186/s12870-014-0235-1)
Supplement: Additional file 2: — Effect of different kanamycin concentrations on regeneration from hypocotyl, cotyledonary node and cotyledon explants. [file 12870_2014_235_MOESM2_ESM.doc]

**Additional file 2 Effect of different kanamycin concentrations on regeneration from hypocotyl, cotyledonary node and cotyledon explants**

|  | Percentage of explants with green regenerating shoots | | | |
| --- | --- | --- | --- | --- |
|  | 0 mg·L^-1^ | 10 mg·L^-1^ | 25 mg·L^-1^ | 50 mg·^-1^L |
| Hypocotyl | 100% | 35% | 0% | 0% |
| Cotyledonary node | 100% | 17% | 0% | 0% |
| Cotyledon | 100% | 19% | 0% | 0% |

Note: the explants are from 7-days-old in vitro seedlings, as previously described.
